# Editorial: *Burkholderia* spp.-transmission, pathogenesis, host-pathogen interaction, prevention and treatment

**DOI:** 10.3389/fmicb.2023.1226865

**Published:** 2023-06-06

**Authors:** Silvia Buroni, Ramar Perumal Samy

**Affiliations:** ^1^Department of Biology and Biotechnology “Lazzaro Spallanzani”, University of Pavia, Pavia, Italy; ^2^Department of Anatomy, Yong Loo Lin School of Medicine, National University of Singapore, Singapore, Singapore

**Keywords:** *Burkholderia* spp.-transmission, host-pathogen interaction, prevention and treatment, multidrug resistance, prevention

Bacteria belonging to the *Burkholderia* genus are rod-shaped, Gram-negatives, non-spore-forming, obligate aerobics. They can be found in the environment and are able to cause infections in plants and in animals, where they affect especially immunocompromised patients (Rodríguez-Cisneros et al., 2023). Within this Research Topic, different aspects related to *Burkholderia* spp. were investigated and discussed.

One of the main problems is related to the treatments that can be used to cure the infections caused by these bacteria. Indeed, most strains are multidrug resistant (MDR) (Mahenthiralingam et al., 2008), and this poses a serious threat in the management of infections. MDR phenotypes have been mainly ascribed to class A β-lactamases (Rhodes and Schweizer, 2016), penicillinase with broad spectrum activity (Hwang and Kim, 2015), efflux pump over-expression (Perrin et al., 2010; Bazzini et al., 2011), mutations in the drug target and reduced permeability (Scoffone et al., 2017).

Pattinson et al. used a multi-omic approach to investigate the mechanism of action of 12- cationic bola-amphiphile, 12,12′-(dodecane-1,12-diyl) bis (9-amino-1,2,3,4-tetrahydroacridinium), named 12-bis-THA against *Burkholderia thailandensis*. They compared the efficacy of the compound against capsulated and unencapsulated strains and no differences were observed in the minimum inhibitory concentration values. On the other hand, time-kill analysis revealed that the unencapsulated strain was more susceptible. So the authors decided to use proteomic and metabolomic tools to better understand how the compound works. Their findings suggest that 12-bis-THA affects the central metabolism and suppresses the production of the F1 domain of ATP synthase.

An alternative to fight drug resistance is represented by bioactive compounds, which are basically secondary metabolites (Elshafie et al., 2017). Natural products have higher chemical novelty than that synthetic drugs and are proving a boon in pharmaceutical industries together with molecular informatics, which has come up as an important tool in finding the relationships between molecules and their biological effects (Bender and Glen, 2004). The study by Prasad et al. is based on the screening of soil pathogens exhibiting strong antimicrobial activity, identifying bioactive compounds, elucidating the mode of action of compounds in an antimicrobial assay using *in silico* techniques, and identifying the likely targets through molecular docking studies. A total of 53 bacterial isolates were screened for their antimicrobial potential: the strains JRBHU6, which showed the highest antimicrobial property, was identified as *Burkholderia seminalis*. The strain produced hydrolytic enzymes (chitinases/cellulose) of significance in accrediting its antimicrobial effects. The bioactive metabolites produced by the isolate were extracted in different organic solvents and exerted the most potent growth inhibitory effects against multi-drug resistant *Staphylococcus aureus* and against fungal strains such as *Fusarium oxysporum, Aspergillus niger, Microsporum gypseum, Trichophyton mentagrophytes, Trichoderma harzianum*. This bioactive metabolite was identified as pyrrolo (1,2-a)pyrazine-1, 4-dione, hexahydro, pyrrolo(1,2-a)pyrazine-1,4-dione, hexahydro-3(2-methylpropyl).

Another important point to find alternative solutions to treat *Burkholderia* infections is to fully understand the immune response triggered by the presence of bacteria.

The study by Zhang et al. investigated the adaptive immune responses of dairy cows suffering from mastitis caused by *Burkholderia contaminans*. They mainly used flow cytometry to evaluate T- and B-lymphocytes-mediated immune responses in cows infected by *B. contaminans* and compared it with healthy animals. A significant a significant increase was observed in IgG+CD27+ B lymphocytes, in γδ T cells and double positive CD4+CD8+ T cells and the relative cytokines, and in activated WC1+ γδ T cells in the *B. contaminans* infected cows, indicating a major role of these cells against *B. contaminans* infection. On the contrary, the decreased number of B lymphocytes in infected animals suggests that humoral immune response may not be adequate to fight intracellular infections. All together these results shed light on the immune response triggered by the presence of *Burkholderia* bacteria in animals: this is important especially for the development of a vaccine, which is still lacking, that could help to prevent these infections thus avoiding the overuse of antibiotics in cattle that strongly contributes to the insurgence of MDR strains.

Other two articles of this Research Topic focused on *Burkholderia pseudomallei*, the causative agent of melioidosis. Melioidosis is an important seasonal infectious disease in tropical as well as sub-tropical counties (Suputtamongkol et al., 1994; Liu et al., 2015).

In the first study, the regulation of virulence factors involved in surface attachment was studied (Sun et al.). Different techniques were used, including high-throughput transposon mutagenesis screening, RNA-Seq analysis, qRT-PCR, and the results revealed that the protein SapR is a transcriptional regulator that activates the surface attachment protein Sap1, as well as genes associated with the bacterial membrane in response to diverse environments, including those encoding virulence factors important for the intracellular survival of *B. pseudomallei*.

In several parts of the world, including South West India, most of the clinical cases of human infection are reported during the time of heavy rainfall. However, the underlying root causes of this phenomenon are not clearly understood yet. India is one of the counties with the highest predicted cases of melioidosis burden worldwide. Surprisingly, there is very little information on the environmental distribution of *B. pseudomallei* as well as its main determining factors.

The study by Shaw et al. clearly reported that the prevalence of *B. pseudomallei* found in soil, especially in South West India, was determined by geochemical factors. They collected a total of 1,704 soil samplings from 20 diverse agricultural sites, particularly during the monsoon as well as post-monsoon season/summer, and screened them by quantitative real-time PCR (qPCR). Results of direct qPCR detected *B. pseudomallei* in all 20 sites. Further, *B. pseudomallei* DNA-positive samples were negatively linked with the concentration of iron, and manganese/nitrogen. The study clearly proves that South West India showed a wide environmental distribution of *B. pseudomallei* but also considerable differences in the abundance between sites and within single sites. In addition, nutrient-depleted habitats promote the presence of *B. pseudomallei*. Most importantly, the study confirmed that the very high levels of *B. pseudomallei* abundance in the soil are found during the rainy season. Indeed, most of the cases were clinically diagnosed during the monsoon season (Vidyalakshmi et al., 2012; Mukhopadhyay et al., 2018).

## Author contributions

Both authors listed have made a substantial, direct, and intellectual contribution to the work and approved it for publication.
